# Renal pelvic rupture: A case report of an unexpected cause

**DOI:** 10.1016/j.ijscr.2021.106176

**Published:** 2021-07-07

**Authors:** Jaafar Fouimtizi, Abdelmoughit Hosni, Laila Jroundi, Amine Slaoui, Abdellatif Koutani, Ahmed Ibn Attya Andaloussi

**Affiliations:** a Department of urology “B”, Ibn Sina Universitary Hospital, Rabat, Morocco; b Department of Emergency Radiology, Ibn Sina Universitary Hospital, Rabat, Morocco; c Faculty of medicine and pharmacy of Rabat, University Mohamed V, Rabat, Morocco

**Keywords:** CT, Computed tomography, MR, Magnetic resonance, T1W, T1 weighted sequence, T2W, T2 weighted sequence, FatSat, Saturation of fat, FIESTA, Fast Imaging Employing Steady-state Acquisition, DWI, Diffusion weighted imaging, UTUC, Upper tract urothelial carcinomas, UC, Urothelial carcinomas, Case report, Renal pelvis, Rupture, Ureteral tumor, Urinoma, Hematoma

## Abstract

**Introduction:**

Renal pelvic rupture (RPR) is a rare condition, that is most usually caused by obstructive calculi.

In another hand, primal ureteral tumors are also uncommon, with only a few cases reporting their involvement in a RPR.

**Presentation of case:**

We report a case with a multimodality discussion of an ureteral tumor, with a spontaneous renal pelvic rupture (RPR) forming a large retrorenal urohematoma.

**Discussion:**

Only few series reported the subject of RPR in the English literature. Only some single cases reported the causality of urinary tract tumors in RPR.

RPR is an imaging based diagnosis. Herein, upper urinary tract tumors show a variable appearances at imaging.

**Conclusion:**

By reporting this case, we highlight the role of both computed tomography (CT) and magnetic resonance (MR) imaging in the diagnosis of the RPR and their accuracy in the detection of the ureteral tumor. We also consolidate the effectiveness of the conservative attitude in the management of the RPR.

## Highlights

• An ureteral tumor can lead to a renal pelvic rupture.
• Both CT and MR imaging are accurate in the detection of the ureteral tumors.
• Non-surgical management of renal pelvic ruptures is efficient.

## 1 Introduction

Renal pelvic (or forniceal) rupture (RPR) is a rare condition in clinical practice. It is most usually caused by obstructive calculi. Other causes include ureteral extrinsic compression by both malignant or benign neoplasms, pregnancy, iatrogenicity or traumas.

In another hand, primal ureteral tumors are uncommon, representing less than 5% of all urothelial carcinomas. However, there's only a few cases reporting their involvement in a RPR [1].

We report a case of a multimodality discussion of a rare presentation of an ureteral tumor, with a spontaneous rupture of the renal pelvis forming a large retrorenal urohematoma.

The case reported was managed by the authors in an academic hospital and written in line with the SCARE criteria [2].

## 2 Case presentation

A. K., a 48 y.o. male presented by himself to the emergency department with a history of macroscopic hematuria and lower back pain evolving for 2 months. The patient was a chronic smoker with a history of 20 pack-years. He reported an estimated weight loss of 10 kg in 6 months and denied any history of trauma, previous surgery, urolithiasis or past illnesses. He was pale, eupneic, afebrile with a tachycardia at 90 bpm. No other evidence was revealed on the physical examination. A blood sample was taken and the blood count showed an hypochromic microcytic anaemia with an heamoglobin level at 8,7 g/dL. The serum renal tests were at normal values (creatinimea: 8.8 mg/L; Urea: 0.42 g/L).

**Fig. 1:**
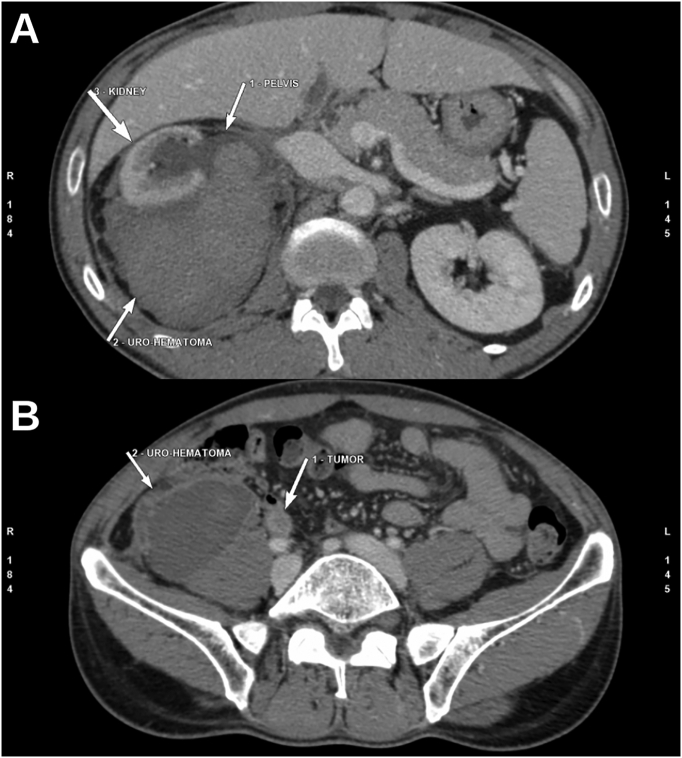
Abdominal CT images at the parenchymal phase: (A) A retrorenal collection showing a mixed liquid and blood densities; (B) The retrorenal collection is extending to pelvic fossa with the visualization of an enhancing lesion at the lumbar ureter.

The patient was hospitalized and a contrasted CT-Urogram (Fig. 1) was requested as an investigation for the heamaturia. A massive right retrorenal collection was found with a mixed areas of fluid and blood densities. The renal pelvis was dilated with a lack of renal excretion at the late phase. In parallel, at the parenchymal phase, we noted a slight contrast enhancing lesion in the lumbar ureter, measuring 11 × 8 × 10 mm.

**Fig. 2:**
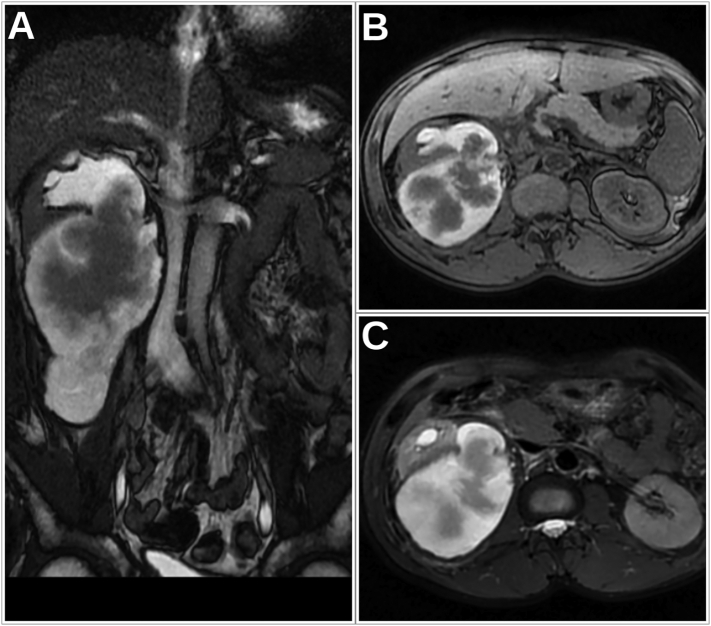
Abdominal MRI images: (A) A coronal FIESTA sequence showing the retrorenal collection emerging at the posterior wall of the pelvis; (B,C) An axial T1W and T2W with FatSat showing the hemorrhagic signal (high signal in both T1W and T2W FatSat sequences) in both the collection and the renal pelvis.

**Fig. 3:**
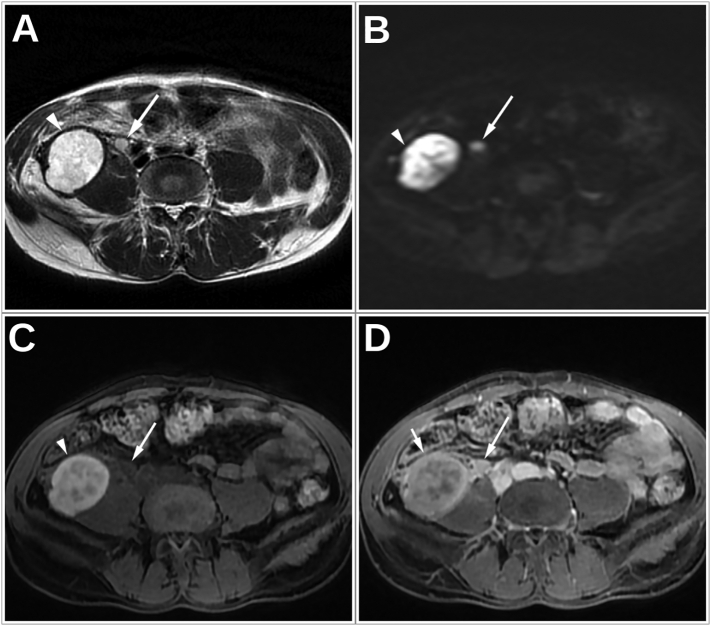
Abdominal MRI images at T2W (A), DWI (B), T1W FatSat before (C) and after (D) gadolinium administration: The ureteral lesion (Arrow) is showing a high signal on both T2W and DWI sequence with a low signal at T1W and an enhancement after Gadolinium administration.

An MR-Urogram was performed for a further characterization. The signal on the retrorenal collection was both liquid and hemorrhagic (Fig. 2). The renal pelvis was dilated with a hemorrhagic content. Indeed, the ureteral lesion was evident (Fig. 3) as a high signal on T2W with a diffusion restriction and enhancement on Gadolinium based sequences.

The patient benefited from a flexible ureteroscopic examination confirming the ureteral lesion and guiding biopsies. Herein, the pathological examination confirmed the diagnosis of a papillary urothelial carcinoma, grade 2.

We discussed the management options for this patient, while no evidence of lymph node enlargement or metastatic lesions were noted on previous imaging. The patient elected to undergo a laparoscopic radical nephroureterectomy with a bladder cuff excision. He was operated by our team, 3 weeks after his first presentation. With the final pathological evaluation, the disease was staged pT2N0M0.

As an adjuvent therapy, a 21-day cycles of cisplatin and gemcitabin based chemotherapy were managed after three months from the surgical procedure. The patient had no further hematuria. Furthermore, serial follow-ups during one year, using cystoscopy and CTs showed no evidence of a disease recurrence.

## 3 Discussion

Renal pelvic rupture (RPR) is an imaging based diagnosis with a perirenal urine extravasation. Causes are mainly obstructive and are dominated by obstructive calculi, which are reported in around 80% of cases. Only few series reported the subject in the English literature. Morgan et al. listed malignancies as the second most common cause of RPR. However, they didn't define as if it concerns only urinary tract malignancies or even extrinsic malignancies causing compression on the urinary tract. In another series by Gershman et al., they didn't cite any case of primal urinary tract tumor, while extrinsic ureteral compressions were listed as the second cause of RPR [3], [4]. Otherwise, only some single cases reported the causality of urinary tract tumors in RPR [1].

In another hand, tumors in the upper urinary tract (ureters and/or pyelocaliceal cavities) are dominated by urothelial carcinomas (UC). Upper tract urothelial carcinomas (UTUC) are rare, accounting for only 5–10% of all UC, while the bladder accounts for 90–95%. We should say that UC is an urothelial disease with a great association between UTUC and bladder tumors [5]. This coincidence didn't occur in our patient in the limited follow-up duration of one year.

UTUC may be incidentally found on imaging. Otherwise, haematuria is the most common appealing symptom. Other symptoms include flank pain, or systemic symptoms, as fatigue, weight loss… The latter should raise the attention for an advanced/metastatic disease [5].

At imaging, CT-Urography (CTU) is the most accurate technique in the diagnosis and staging of UTUC. Its rapid acquisition of a thin slices, offering an isotropic resolution, allows an accurate visualisation of the tumor in multiple planes. MR-Urography is less accurate, and is used mainly in cases of contraindications to CT [5], [6]. We conducted an MR-Urography in our patient to better characterize the content of the perirenal collection, while no active extravasation of contrast was shown on CT. Then, MRU, with its high contrast resolution, showed the communication between the collection and pelvis confirming the RPR.

Otherwise, ureteral tumors may appear as a sessile or polypoid filling defect, a focal eccentric or circumferential wall thickening or an infiltrative ureteral and periureteral mass [6]. The optimal opacification and distension of the urinary cavities condition the performance of the imaging techniques. In the case we reported, both techniques showed the ureteral lesion as an obstructive polypoid filling defect. The lesion was enhancing after contrast injection, allowing to distinguish the tumoral lesion from a blood clot.

There's no consensus about how to manage RPR. Treatment may be non-surgical with or without antibiotics administration; or surgical using ureteral stenting or nephrostomy. Morgan et al. argued that non-surgical management should be privileged. Herein, they suggested that the place of antibiotic therapy and surgery should be ruled by arguments on infection. These latter include fever, leukocytosis, positive urinalysis, or elevated creatinine [3].

Finally, in a context of UTUC, hydronephrosis, which is the mechanism that leads to RPR, places the patient at a “high-risk” group, making him a bad candidate to kidney-sparing surgery [5].

## 4 Conclusion

Besides its rarity and the usual association with urolithiasis, a renal pelvic rupture can unmask an upper urinary tract malignancy. We should say that imaging is accurate in the determination of UTUC, with a variable appearances. The non-surgical attitude of RPR is effective to save time for a carcinologic planning and management.

## Funding

The authors didn't receive any funding.

## Ethical approval

Ethics approval was given.

## Consent

The consent to participate was given by the patient included in the article.

## Registration of research studies

This article does not concern a “First in Man” study.

## Guarantor

Jaafar Fouimtizi M.D.

## CRediT authorship contribution statement

All authors contributed equally to this work. All authors read and approved the final manuscript.

## Declaration of competing interest

No conflict of interest.
